# Chondroma of the scalp

**DOI:** 10.11604/pamj.2015.21.64.7067

**Published:** 2015-05-28

**Authors:** Rachid Ammor, Assou Ajja

**Affiliations:** 1 Military Hospital My Ismail, Department of Neurosurgery, Meknes, Morocco

**Keywords:** Chondroma, scalp, nodule

## Image in medicine

A 26 year old man, with no specific past or family history, presented with slow growing subcutaneous mass in the right parietal region of about 4 cm. Skin examination revealed a firm subcutaneous nodule that was movable over the underlying bone. Physical examination was not remarkable other than above-described skin lesion. X-rays of the skull (A) and CT scan (B) showed a subcutaneous mass next to the right parietal eminence measuring 17×39 mm and containing calcifications. Surgery resection of the tumor was complete and easy (C). The nodule was not attached to underlying skull. The postoperative course was unremarkable. Histological examination confirmed the diagnosis of soft-tissue chondroma (D). Extraskeletal chondroma is a rare, benign cartilaginous tumor of the soft tissue. It presents as a solitary subcutaneous mass measuring less than 3 cm in diameter that is usually painless and slowly growing. It is most frequently found in the hands and feet of adults in the fourth and fifth decades. Its location in the scalp is exceptional and atypical. Complete excision is recommended for the treatment of extra skeletal chondroma.

**Figure 1:**
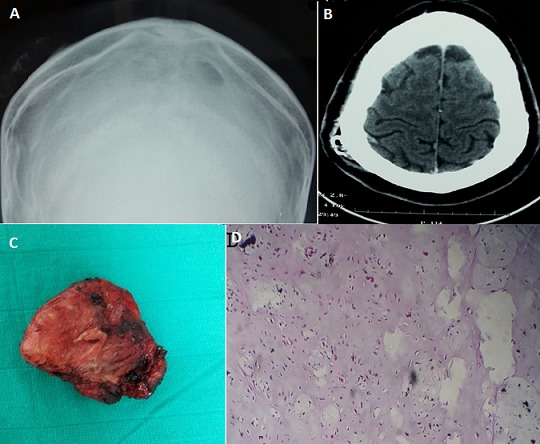
(A) X-rays of the skull showing a subcutaneous tumor in the right parietal region containing calcifications; (B) CT scan showing a nodule of the scalp of about 17×39 mm containing calcifications and without contrast enhancement after injection; (C) the nodule after full excision; (D) the histopathologic study shows a limited cartilage tissue containing mature chondocytes islets. There was no mitosis or atypia

